# Immunodeficiency in Bloom’s Syndrome

**DOI:** 10.1007/s10875-017-0454-y

**Published:** 2017-11-02

**Authors:** Michiel H. D. Schoenaker, Stefanie S. Henriet, Jip Zonderland, Marcel van Deuren, Qiang Pan-Hammarström, Sandra J. Posthumus-van Sluijs, Ingrid Pico-Knijnenburg, Corry M. R. Weemaes, Hanna IJspeert

**Affiliations:** 1 Department of Pediatric Infectious Diseases and Immunology, Radboud University Nijmegen Medical Centre, Nijmegen, The Netherlands; 2 Department of Immunology, Erasmus MC, University Medical Center Rotterdam, Wytemaweg 80, 3015 CN Rotterdam, The Netherlands; 3 Department of Internal Medicine, Radboud University Nijmegen Medical Centre, Nijmegen, The Netherlands; 4 Division of Clinical Immunology and Transfusion Medicine, Karolinska Institutet at Karolinska University Hospital, Huddinge, Stockholm, Sweden

**Keywords:** Immunodeficiency, bloom’s syndrome, lymphocyte, DNA repair, somatic hypermutations, class switch recombination

## Abstract

Bloom’s syndrome (BS) is an autosomal recessive disease, caused by mutations in the *BLM* gene. This gene codes for BLM protein, which is a helicase involved in DNA repair. DNA repair is especially important for the development and maturation of the T and B cells. Since BLM is involved in DNA repair, we aimed to study if BLM deficiency affects T and B cell development and especially somatic hypermutation (SHM) and class switch recombination (CSR) processes. Clinical data of six BS patients was collected, and immunoglobulin serum levels were measured at different time points. In addition, we performed immune phenotyping of the B and T cells and analyzed the SHM and CSR in detail by analyzing IGHA and IGHG transcripts using next-generation sequencing. The serum immunoglobulin levels were relatively low, and patients had an increased number of infections. The absolute number of T, B, and NK cells were low but still in the normal range. Remarkably, all BS patients studied had a high percentage (20–80%) of CD4+ and CD8+ effector memory T cells. The process of SHM seems normal; however, the Ig subclass distribution was not normal, since the BS patients had more IGHG1 and IGHG3 transcripts. In conclusion, BS patients have low number of lymphocytes, but the immunodeficiency seems relatively mild since they have no severe or opportunistic infections. Most changes in the B cell development were seen in the CSR process; however, further studies are necessary to elucidate the exact role of BLM in CSR.

**Electronic supplementary material:**

The online version of this article (10.1007/s10875-017-0454-y) contains supplementary material, which is available to authorized users.

## Introduction

Bloom’s syndrome (BS) is an autosomal recessive disease, caused by mutations in the *BLM* gene located at 15q26. This gene codes for BLM protein, which is a DNA helicase involved in DNA replication and repair. BS is characterized by predisposition to malignancy, prenatal growth retardation, gastro-esophageal reflux, café-au-lait spots, characteristic butterfly-shaped erythema, and immunodeficiency [1, 2]. The immunodeficiency is characterized by low serum immunoglobulins and different infections. Otitis media is a very common infection among BS patients, especially in children. Also, up to 20% of the BS patients had pneumonia. [1] However, the pathophysiology behind the immunodeficiency in BS has not yet been elucidated.

It is known that DNA repair defects can result in immunodeficiency since DNA repair is essential for the development of antigen receptors expressed on B and T lymphocytes. These antigen receptors are formed by recombination of the variable (V), diversity (D), and joining (J) genes on the antigen receptor loci. During this V(D)J recombination process, DNA double-strand breaks (DSBs) are introduced near the V, D, and J genes; the DNA ends are processed and eventually ligated by the non-homologous end-joining (NHEJ) DNA repair pathway [3]. When T and B cells express a functional antigen receptor, also called a T cell receptor (TR) and B cell receptor (BCR), they can migrate to the periphery where they can encounter an antigen [4]. After antigen encounter, B cells can further divaricate their BCR by introducing somatic hypermutations (SHM) or by class switch recombination (CSR). SHM increase the affinity of the BCR for their antigen, and CSR changes the effector function of the secreted BCR, also called immunoglobulin or antibody. Both SHM and CSR rely on DNA repair. SHM is initiated by AID, which deaminates cytosine (C) into uracils (U), creating a mismatch with the guanine (G) on the complementary strand [5, 6]. These U:G mismatches can be resolved using three different pathways: base excision repair (BER), mismatch repair (MMR), or replication. During BER, the U is removed and replaced by a random nucleotide by an error-prone polymerase, resulting in transition and transversion mutations at G/C bases [7, 8]. Mutations at A/T bases can occur via the MMR pathway when multiple bases surrounding the U:G mismatch are removed and filled by the error-prone polymerase eta, which introduces errors at A/T pairs specifically at WA/TW motifs [9–11]. Finally, if the U:G mismatch is not repaired before the DNA is replicated, the U will be recognized as a thymine (T), resulting in a C to T transition mutation. During CSR, AID introduces U:G mismatches in the switch regions upstream of the constant genes in the IGH locus [12]. Subsequently, proteins of the BER pathway introduce DNA DSBs, which can be repaired by NHEJ or by alternative end-joining (A-EJ) [13–17].

BLM is described to have a role in at least two DNA repair pathways involved in lymphocyte development: alternative end-joining and BER [18–20]. In addition, BLM can also stimulate DNA synthesis by pol eta [21]. So far, there is no active role discovered for BLM during V(D)J recombination [22]. In mice, there is a reduced CSR capacity and a shift to microhomology-mediated switch junction formation [23].

The aim of this study is to give insight of the immunodeficiency in BS and to discover the role of BLM in CSR and SHM in humans.

## Methods

### Cell Samples and Flow Cytometric Immunophenotyping

Peripheral blood samples and clinical data were collected from six patients with BS with informed consent and according to the guidelines of the Medical Ethics Committee of the Radboud University Nijmegen medical Center and Erasmus MC Rotterdam. Flow cytometric analysis of peripheral blood for the healthy controls and patient 5 was performed using 6-color labeling as previously described [24]. For patient 1–4 and patient 6, an 8-color protocol was used with the following antibodies: CD24-PB (SN3) (Exbio, Vestec, Czech Republic), CD45-PO (HI30) (Invitrogen), IgD-FITC and IgM-PE (Southern Biotechnologies, Birmingham, AL), IgA FITC (IS1 1-8E10) (Mitenyl Biotech, Bergisch Gladbach, Germany), IgD-biotine (IA6-2) (Biolegend, San Diego, CA), CD45RO-FITC (UCHL1) (DAKO, Agilent Technologies, Glostrup, Denmark), CCR7-PE (Miltenyi Biotech), CD28-PE-Cy7 (CD28.2; e-Bioscience), CD19-PerCP-Cy5 (SJ25C1), CD27-ApC (LL128), CD38-APC-H7 (HB7), IgG-PE (G18-145), CD4-PB (RPA-T4), CD3-PerCP (SK7), and CD8-APC-H7 (SK1) (all from BD Biosciences, CA, USA). The absolute numbers were calculated BD Trucount™ tubes (BD Bioscience). The following CD19+ B cell subsets were defined: transitional B cells as CD24^high^CD38^high^CD27^−^IgM^+^IgD^+^, naïve mature B cells as CD27^−^ CD24^dim^CD38^dim^IgM^+^IgD^+^, natural effector B cells as CD27^+^IgD^+^CD24^dim^CD38^dim^, and memory B cells as CD27^+^ IgD^−^CD24^dim^CD38^dim^. The following CD3+ T cell subsets were defined: CD8+ naïve T cells as CD8^+^CD45RO^−^CCR7^+^CD27^+^CD28^+^, CD8+ central memory T cells as CD8^+^CD45RO^+^CCR7^+^CD27^+^CD28^+^, CD8+ effector memory T cells as CD8^+^CCR7^−^. CD4+ naïve T cells as CD8^−^CD45RO^−^CCR7^+^CD27^+^CD28^+^, CD4+ central memory T cells as CD8^−^CD45RO^+^CCR7^+^CD27^+^CD28^+^, and CD4+ effector memory T cells as CD8^−^CCR7^−^.

### Repertoire Analysis of IGH Transcripts Using Next-Generation Sequencing

PBMC’s were isolated from peripheral blood or cord blood samples using Ficoll. mRNA was isolated using the Gen-Elute Mammalian total RNA miniprep kit from Sigma-Aldrich (St. Louis, MO). cDNA was created from 2 μg RNA using the Superscript II reverse transcriptase kit from Invitrogen (Paisley, UK). IGH rearrangements were amplified in a multiplex PCR using the forward VH1-6 FR1 (BIOMED-2) primers [25] and either the CgCH1 [26] or the IGHA [27] reverse primer. The PCR products were purified and sequenced using Roche 454 sequencing as previously described [28]. In short, PCR products were purified by gel extraction (Qiagen, Valencia, CA) and Agencourt AMPure XP beads (Beckman Coulter, Fullerton, CA). Subsequently, the PCR concentration was measured using the Quant-it Picogreen dsDNA assay (Invitrogen, Carlsbad, CA). The purified PCR products were sequenced on the 454 GS junior instrument according the manufacturer’s recommendations. Sequences were demultiplexed based on their multiplex identifier sequence and 40 nucleotides trimmed from both sides to remove the primer sequence using ARGalaxy [29] (https://bioinf-galaxian.erasmusmc.nl/argalaxy). Fasta files were uploaded in IMGT/High-V-Quest [30], and subsequently the IMGT output files were analyzed in ARGalaxy [29]. Only productive sequences that were complete, without ambiguous bases, present twice or more, in which a C subclass could be defined, were included once in the analysis. All information on the FR1 region was excluded from the analysis since the forward primers used to amplify the transcripts were located in FR1. The age and number of sequences used for the analysis are listed in Supplemental Table 1. The percentage SHM was calculated per sequence by dividing the number of mutations in the CDR1-FR3 region by the number of nucleotides in the CDR1-FR3 region. The CDR3 region was excluded from the SHM analysis since it is not possible to distinguish true somatic mutations from N-nucleotides. In addition, the Immunoglobulin analysis tool (IgAT) [31] was used to determine the percentage of antigen-selected sequences. The data from P1 to P3 were compared to data obtained from six healthy children (7–15 years) and data from P4 to P6 were compared to ten healthy adults (31–55 years).

### Characterization of Switch Recombination Junctions

The Sμ-Sα recombination fragments were PCR amplified from DNA derived from peripheral blood cells and sequenced as previously described [16, 32]. The pattern of CSR junctions was analyzed according to guidelines [33].

### Principal Component Analysis

Z-scores were calculated by the sample score minus the mean of the controls divided by the standard deviation of the controls. Thereby, the mean and standard deviation of either the controls between 7 and 15 years of age (*n* = 6) or the adult controls (*n* = 10) were used. Since we had missing data for either IGHG or IGHA in three controls, we did not plot these controls in the graph. This resulted in 32 parameters for 13 controls and 6 BS patients. Subsequently, components and contributions were calculated and plotted using prcomp in R [34].

## Results

### Patients

Patient characteristics are shown in Table 1. We describe three children (9–12 years) and three adult BS patients (36–49 years). The adult BS patient P4 (SuS or SuSc), P5 (MP or MaPa), and P6 (GC) have been described previously when they were in their childhood [35, 36]. All patients have a low birth weight, and birth length indicating a growth deficiency. The characteristic butterfly-shaped erythema was present in all patients. Recurrent ear infections were present, which is a well-known phenomenon in BS [1]. Two adult patients had a double solid malignancy, both were treated with success. Patient 5 was diagnosed with a monoclonal gammopathy of unknown significance (MGUS), which did not deteriorate in a multiple myeloma so far. In addition, patients suffered from different infections like bronchitis, uveitis, and two out of five patients had pneumonia. However, the patients in this cohort did not have opportunistic infections, sepsis or other life-threatening manifestations, or a serious failure of the immune system.

**Table 1:** Patient characteristics

Patient	Age at analysis	Mutation	Birth weight	Birth length	Butterfly-shaped erythema	Recurrent ENT infections	Infections	Malignancy	Other
P1	9	c.2695C>T/c.2695C>T	1845	Unknown	+	+	Tonsillitis	–	–
P2	12	c.1933C>T/c.2407-1G>T	2000	45	+	+	Herpes Zoster Urinary tract Respiratory tract Gastro-enteritis Pneumonia Conjunctivitis Rhinitis Paronychia	–	–
P3	12	c.1933C>T/c.1933C>T	1025	34	+		Neonatal toxoplasmosis		Growth hormone replacement therapy
P4	35	c.1284G>A/c.1933C>T	1270	39	+	+	Pneumonia	Squamous cell carcinoma and colon carcinoma	–
P5	37	c.2488_2489dupA/c.3681del	2000	Unknown	+	+	Bronchitis Gastro-enteritis Varicella zoster Urinary tract Uveitis Bacterial conjunctivas	Polyps Mamma carcinoma Adenocarcinoma of the bowel	Monoclonal gammopathy of unknown significance (MGUS) Diabetes Mellitus
P6	46	c.1933c>T/c.1933c>T	1810	48	?	+	Bronchitis	Polyp during colonoscopy	Tonsillectomy Adenotomy Abscesses

### BS Patients Have Subnormal Numbers of Lymphocytes

**Fig. 1:**
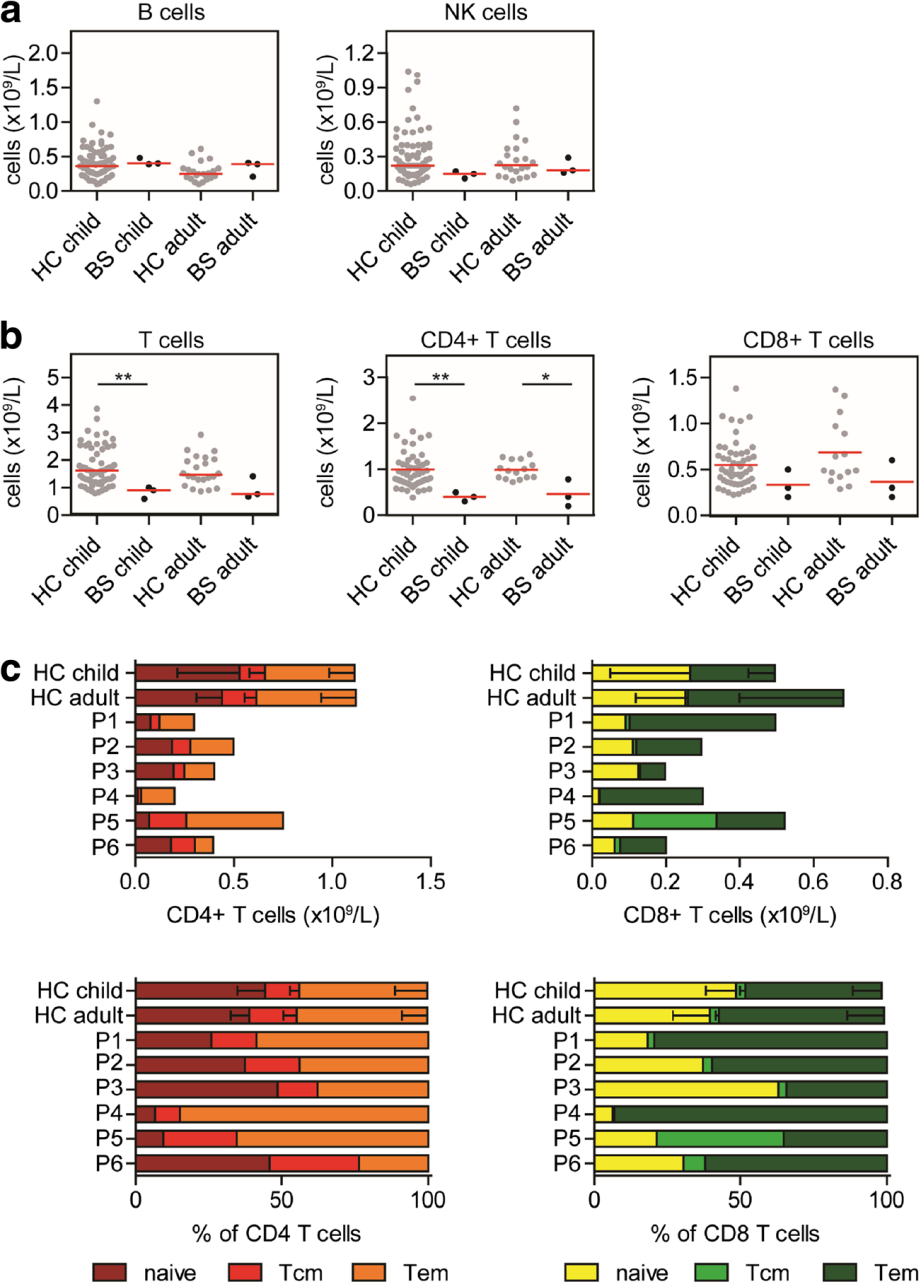
T cell subsets in BS patients. The absolute numbers of CD19+ B and CD16+CD56+ NK cells (A), CD3+ T cells, CD4+ T (CD3+ CD8-) cells, and CD8+ T cells (CD3+ CD8+) (B) are low in the BS patients compared to the healthy controls (HC). [37] The absolute numbers of CD4+ and CD8+ naïve (CD45RO^−^CCR7^+^CD27^+^CD28^+^), central memory (Tcm) (CD45RO^+^CCR7^+^CD27^+^CD28^+^), and effector memory (Tem) (CCR7^−^) are reduced in the BS patients, but the relative distribution is normal in most BS patients (C). Significant values were calculated using the two-tailed Mann-Whitney test and are indicated: *P ≤ 0.05; **P ≤ 0.01

To elucidate the effect of BLM deficiency on the lymphocyte development, we analyzed the lymphocyte subsets using flow cytometry. We compared the absolute numbers of CD3+ T cells, CD4+ T cells, CD8+ T cells, B cells, and NK cells with previously published data from 71 healthy children (5–16 years of age) and 22 healthy adults [37]. The absolute numbers of B- and NK cells in the BS patients were in the range of the healthy controls (HC) (Fig. 1a). The absolute number of T cells was significantly lower in the BS children compared to HC, and low in the adult BS patients (Fig. 1b). The CD4+ T cells were significantly reduced in all BS patients, while the CD8+ T cells were low but in the normal range. The absolute number of CD4+ and CD8+ naïve, effector memory (Tem), and central memory (Tcm) populations were reduced compared to the HC (Fig. 1c). However, the distribution of the naïve, Tem, and Tcm populations was relatively normal in most of the BS patients, except for P4 who had a strong decrease in CD4+ and CD8+ naïve T cells (Fig. 1c). Together, these data showed that BS patients had subnormal levels of T cells.

### Decreased Memory B Cells and Subnormal Levels of Immunoglobulins

**Fig. 2:**
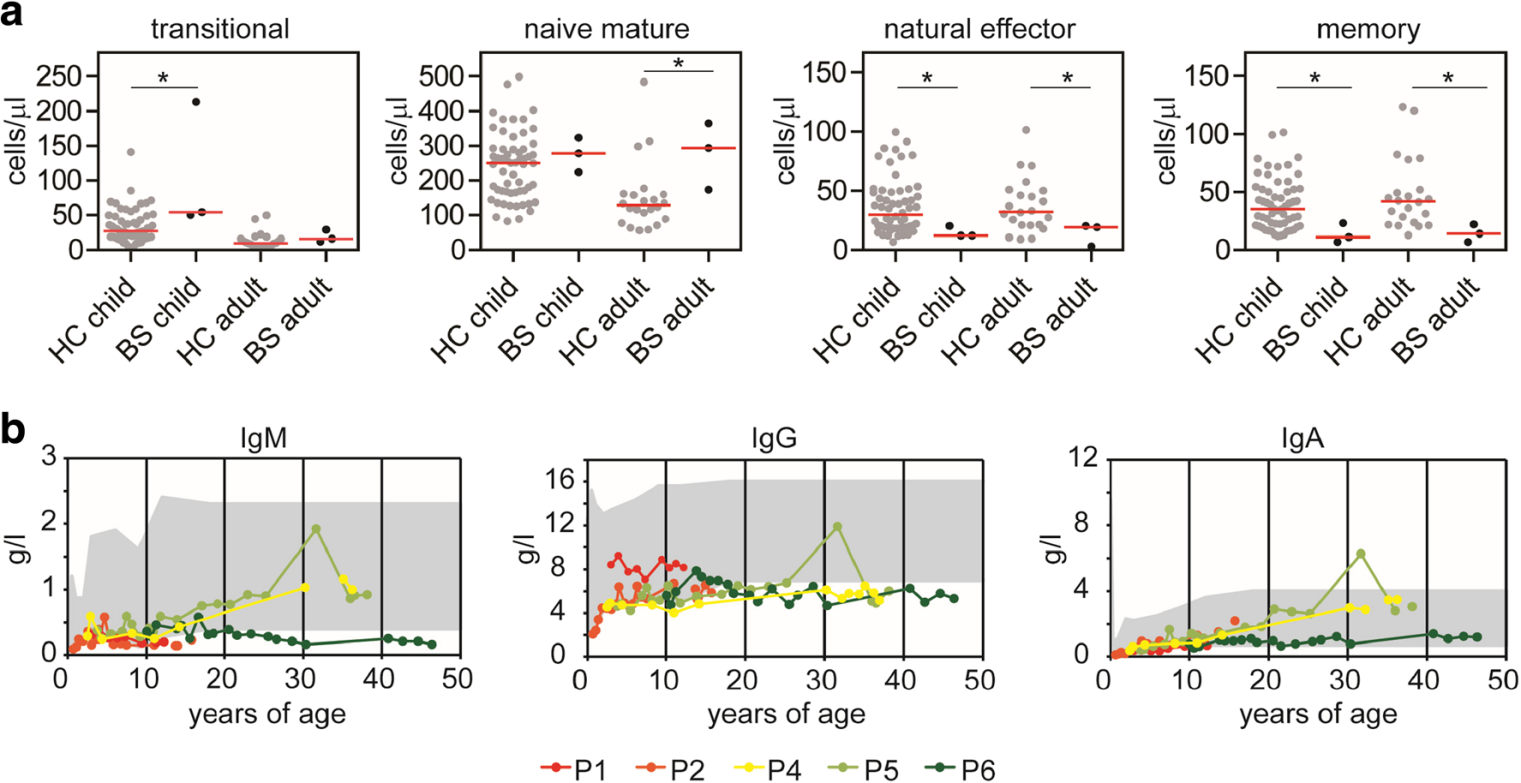
B cells and serum immunoglobulin levels. The absolute numbers of transitional (IgD^+^CD27^−^CD24^++^CD38^++^) and naïve mature B cells (IgD^+^CD27^−^CD24^+^CD38^+^) are normal or increased in the BS patients compared to the age-matched healthy controls (HC), while the natural effector (IgD^+^CD27^+^IgM^+^) and memory B cells (IgD^−^CD27^+^) are decreased (**a**). The serum IgM, IgG, and IgA immunoglobulin levels are persistently low in most of the BS patients. The gray area indicates the range of the reference values. Significant values were calculated using the two-tailed Mann-Whitney test and are indicated: *P ≤ 0.05

While the total number of B cells were in the normal range, the absolute number of transitional and naïve mature B cells was normal or slightly increased, and natural effector B cells and memory B cells decreased compared to HC (Fig. 2a). Since this suggests that B cell maturation might be impaired in the final stages of differentiation, we analyzed the levels of serum immunoglobulins over the past decades (0–40 years of age). The IgM, IgG, and IgA levels were all low but mostly at the border of the normal range during the years (Fig. 2b). Only P5 has a relative peak in both IgG, IgM, and IgA at 31 years of age, which is unexplained so far (Fig. 2b) The low concentration of total IgG was not caused by a decrease in one of the IgG subclasses, since all the serum IgG subclasses were at the lower border of the normal range (Supplemental Fig. 1).

### Frequency and Repair of SHM Is Normal in the BS Patients

**Fig. 3:**
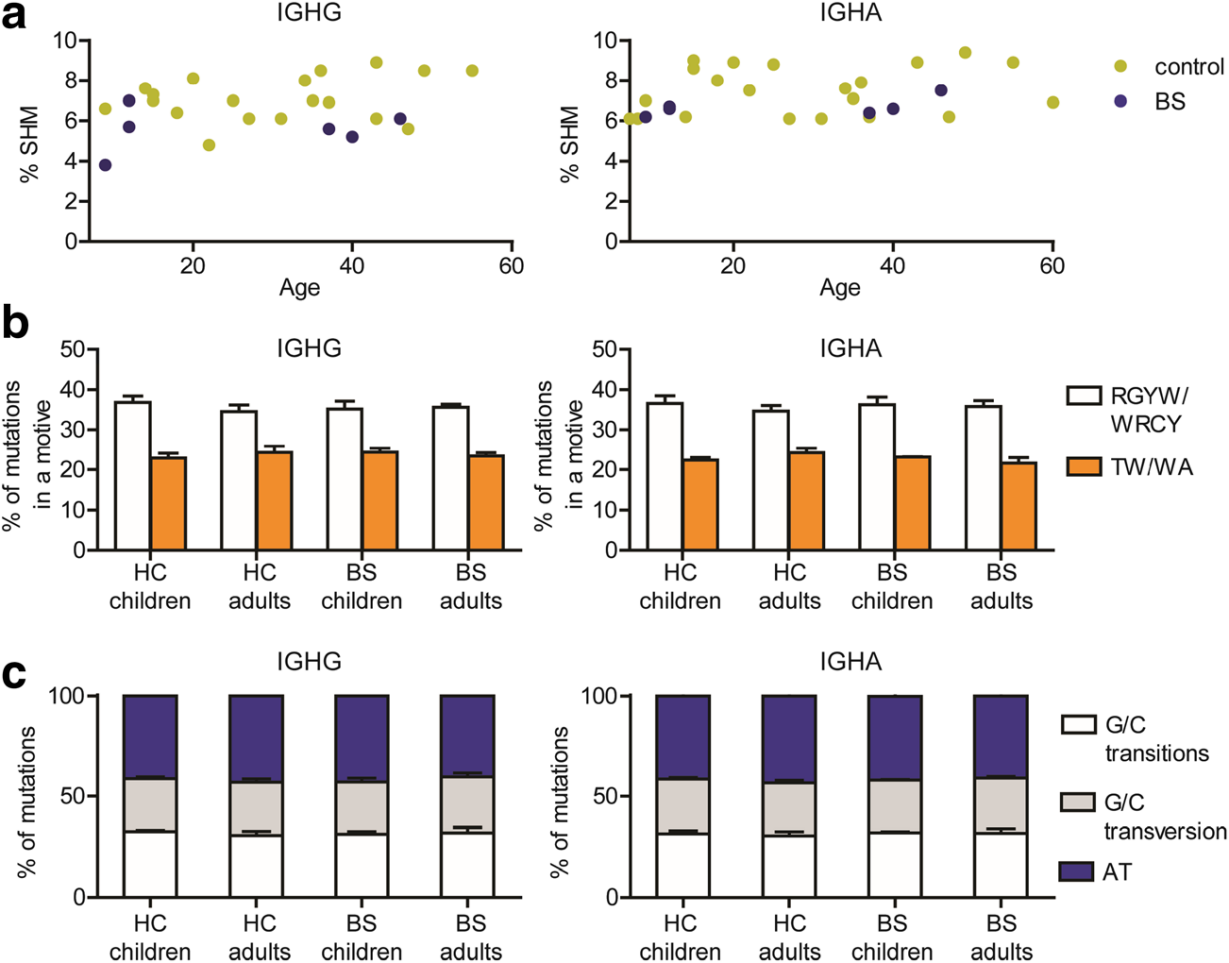
SHM frequency and patterns. Frequency of SHM in IGHG and IGHA transcripts are low but in the normal range in the BS patients (**a**). The mean percentage of mutations located in RGYW/WRCY motives and TW/WA motives (**b**), and the mean percentage of mutations at A/T or G/C (divided in transitions and transversions) locations (**c**) is normal in BS patients. The error bars indicate the standard deviation

Since BLM is involved in BER and has been described to stimulate DNA syntheses by pol eta, we analyzed the frequency and repair patterns of SHM in switched B cells. We used next-generation sequencing to sequence IGHG and IGHA transcripts from the six BS patients and compared the data to age-matched controls (see Supplemental Table 1 for overview for the number of sequences used for the analysis). The median frequency of SHM in both IGHG and IGHA transcripts was in the normal range of the age-matched healthy controls (Fig. 3a). Subsequently, we studied the location and repair patterns of the SHM, which provide information about the repair pathway that is used to introduce the mutation during SHM. Similar to the healthy controls, around 35% of the SHM were located in RGWY/WRCY (AID) and around 20% in WA/TW (pol eta) motif, indicating that the targeting of SHM is not aberrant in the BS patients. Then we investigated the repair patterns of the SHM in more detail. U:G mismatched resolved by the MMR pathways lead to mutations at A/T location, and BER is the dominant pathway for transversion mutations at G/C locations. In the BS patients, 39–47% of the mutations were located in A/T locations, which is comparable to the HC. Moreover, the percentages of transitions and transversions at G/C location in the BS patients were also within the normal range. This indicated that the targeting and repair mechanisms in SHM are not affected by the BLM deficiency.

### Class Switching to more Downstream Constant Regions Is Reduced in BS

**Fig. 4:**
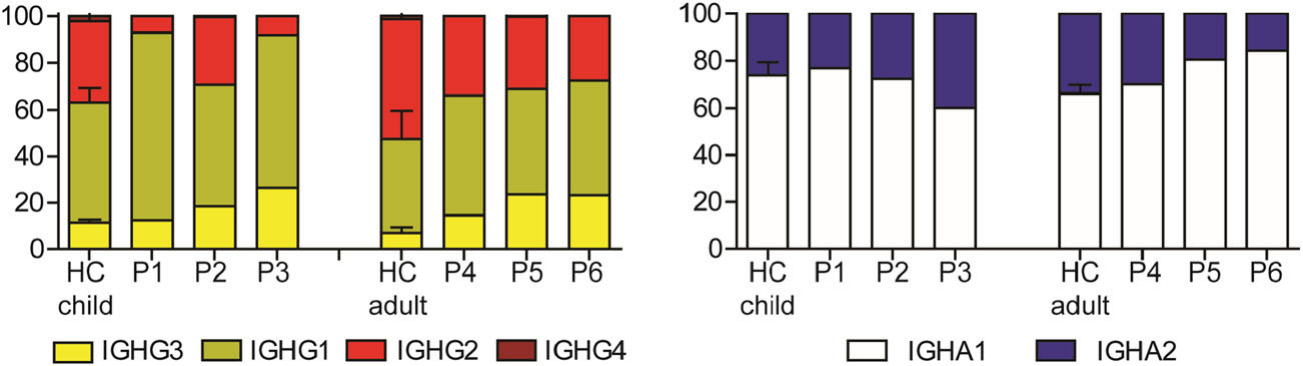
Subclass distribution of the IGH transcripts. BS patients have relatively more IGHG1 and IGHG3 transcripts compared to age-matched controls (**a**). The distribution of the IGHA1 and IGHA2 transcripts is normal (**b**). The bar graphs of the controls indicate the mean and standard deviation

Besides V(D)J recombination and SHM, DNA repair is also crucially important for CSR. We determined the percentages of rearrangements having a certain Cg or Ca constant gene. For both children and adult BS patients, the frequency of IGHG transcripts with Cg1 and Cg3 constant genes were increased compared to age-matched controls. These data suggests that switching to the downstream Cg2 and Cg4 constant genes is reduced in the BS patients. The frequency of Ca1 and Ca2 constant genes was normal in the BS children, but the adults have a slightly higher frequency of Ca2 (Fig. 4).

Since these data indicated that CSR might be hampered in BS patients, we analyzed the repair of the switch junction to determine if BLM has a role in CSR. During CSR, AID deaminates multiple cytosines in the switch regions upstream of the constant genes. Since the switch regions contain many repeats, the DSBs are often repaired using small homologous region (microhomology). The BS patients had a significant increase of 7–9 microhomology in the switch junction; however, the average microhomology use was not different in the BS patients we analyzed (Table 2).

**Table 2:** Characterization of Sμ-Sα junctions

ID	0 bp	Insertions	1–3	4–6	7–9	≥ 10	Total no. of junctions
P1 (9 years)	1 (6%)	3 (18%)	5 (29%)	2 (12%)	5 (29%)*	1 (6%)	17
P2 (12 years)	3 (30%)	1 (10%)	3 (30%)	1 (10%)	2 (20%)	0 (0%)	10
P4 (35 years)	3 (25%)	1 (8%)	2 (17%)	4 (33%)	1 (8%)	1 (8%)	12
P5 (37 years)	2 (12%)	3 (18%)	4 (24%)	1 (6%)	4 (23%)	3 (18%)*	17
P6 (46 years)	2 (13%)	2 (13%)	5 (33%)	1 (7%)	3 (20%)	2 (13%)	15
All BS patients	11 (15%)	10 (14%)	19 (27%)	9 (13%)	*15 (21%)§#*	7 (10%)	71
Controls (child)	31 (17%)	42 (23%)	36 (20%)	29 (16%)	19 (10%)	26 (14%)	183
Controls (adult)	41 (16%)	56 (22%)	91 (36%)	29 (11%)	25 (10%)	14 (5%)	256

The switch junctions were compared with age-matched controls. P1 and P2 with controls (child) and P4, P5, and P6 with controls (adult). Combined data from all BS patients was both compared with controls (child)^§^ and controls (adult)^#^. Italic numbers are significantly different from the age matched controls

*P < 0.05; §*P* < 0.05; #*P* < 0.05

### Selection of the BCR Repertoire Is Subnormal in BS Patients

**Fig. 5:**
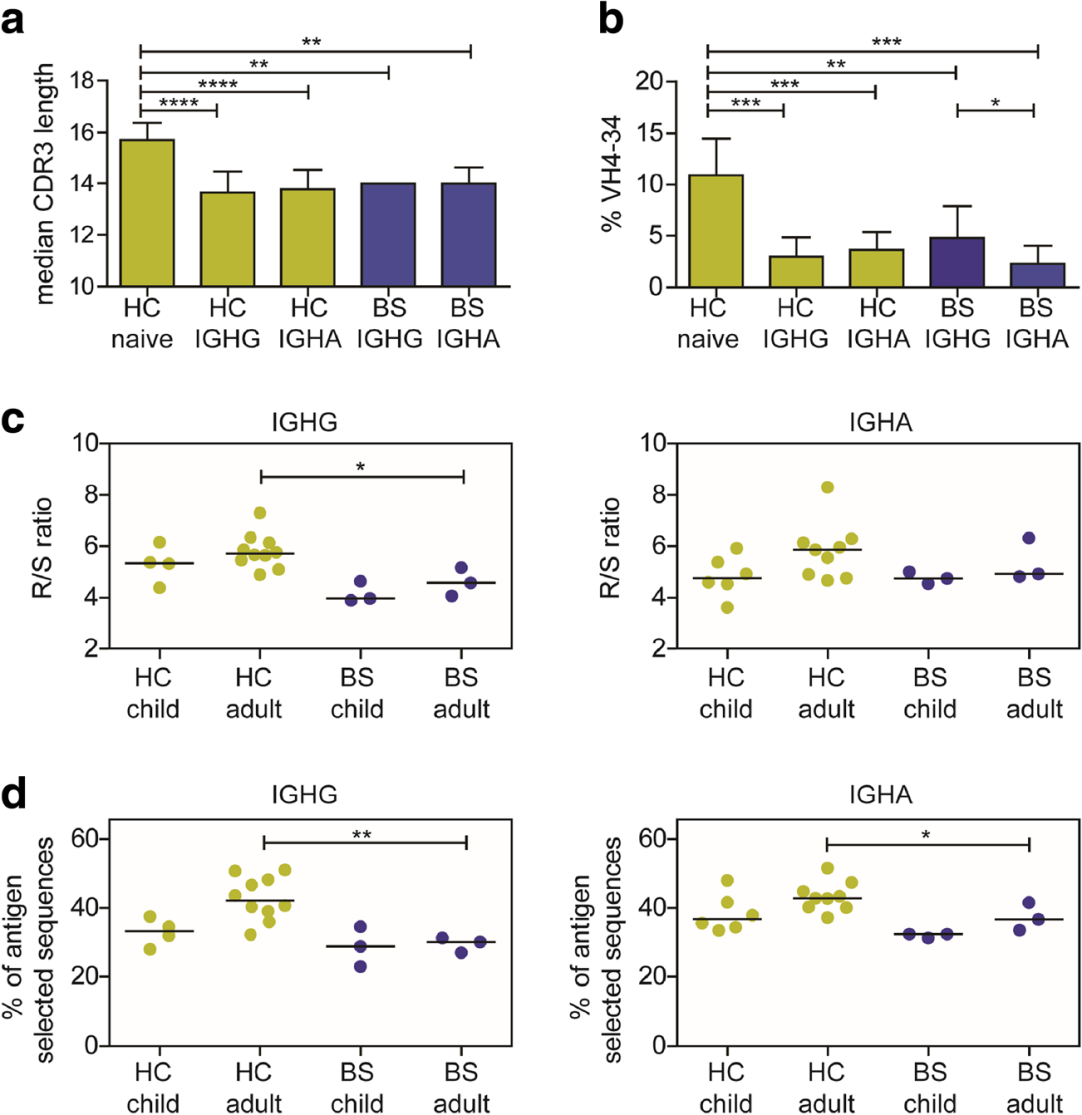
Selection characteristics of the IGH transcripts. The median CDR3 length of IGHG and IGHA transcripts in BS patients is similar to IGHG and IGHA transcripts from healthy controls and significantly shorter than the CDR3 length of naïve B cells (**a**). In the BS patients, the mean percentage of rearrangements using VH4-34 is lower compared to naïve B cells from HC, similarly to switched B cells from the HC (**b**). The replacement/silent (R/S) ratio in the CDR3 regions of the VH genes is lower in the BS patients (**c**). The percentage of antigen-selected sequences as determined using IgAT is lower in the BS patients (**d**). All bar graphs represent the mean and standard deviation. The line in the dotplots represents the median. All columns were compared to the control naïve column, and significant values were calculated using the two-tailed Mann-Whitney test and are indicated: *P ≤ 0.05

Previous studies have shown that the antigen-experienced B cells have shorter CDR3 length and express less often a rearrangement with the IGHV4-34 gene compared to naïve B cells [38, 39]. Similar to HC, the CDR3 length was shorter and the IGHV4-34 usage was reduced in the IGHG and IGHA transcripts derived from the BS patients compared to IGH rearrangements derived from naïve B cells from HC (Fig. 5a and b). In addition, to these changes, BCR rearrangements from antigen-experienced B cells are selected for replacement mutations in the CDR3 region, since they can increase the affinity for their antigen, and against replacement mutations in the FR regions since they can negatively influence the stability of the antibody protein. Therefore, we compared the replacement/silent (R/S) mutation ratio in the IGHG and IGHA transcripts of the BS patients with healthy controls. The R/S ratio seemed lower in the IGHG transcripts but in the normal range in the IGHA transcripts (Fig. 5c). Additionally, we also used the Immunoglobulin Analysis tool (IgAT) which calculates for each rearrangement whether it has undergone antigen selection based on the number of replacement mutations in relation to the total number of mutations. The BS patients had a lower frequency of antigen-selected sequences compared to the age-matched controls, which was significant in the adult BS patients (Fig. 5d). These data suggest that antigenic selection of the B cells is affected by the BLM deficiency.

### BS Patients Cluster Differently from Healthy Controls

**Fig. 6:**
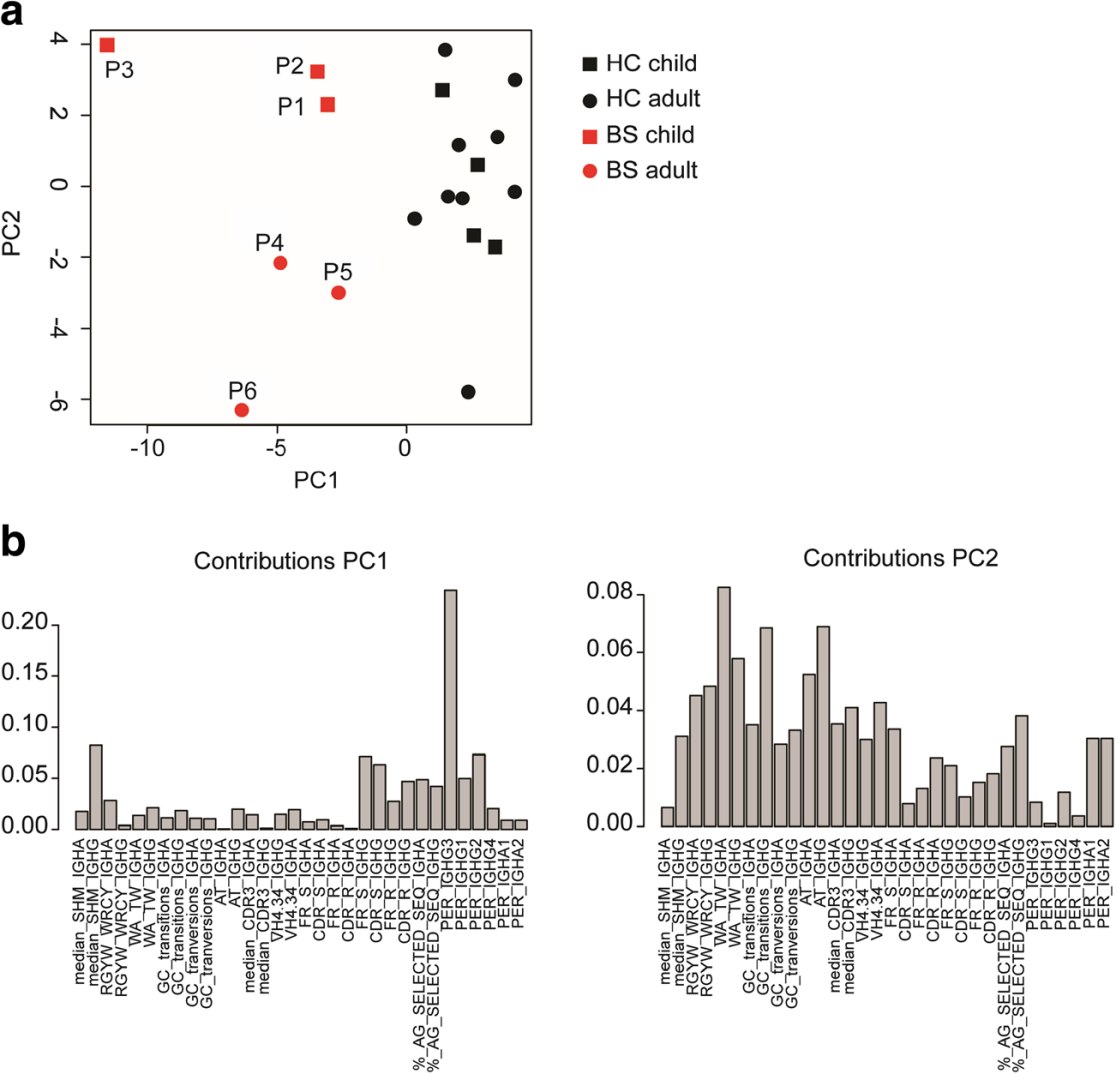
Principal component analysis on the B cell repertoire characteristics. Principal component analysis (PCA) on the data obtained from the IGHG and IGHA transcripts including information on the SHM, subclass distribution, and antigen selection (**a**). Graphs showing how much each variable contributed to PCA1 and PCA2

The data obtained from the IGHG and IGHA transcripts showed that BS patients have small changes in the subclass distribution (Fig. 4) and antigen selection (Fig. 5). To see if the BS patients deviate from the HC, we performed a principal component analysis (PCA) on all the data obtained from the IGHG and IGHG transcripts as displayed in Figs. 3, 4, and 5. This analysis showed that the HC cluster together and that the BS patients indeed separate from the HC (Fig. 6a). The contribution of each parameter of principal component 1 (PCA1) and PCA2 are shown in Fig. 6b. So although we did not find major differences in the B cell repertoire of the BS patients, their repertoire characteristics are clearly deviate from HC.

## Discussion

Immunodeficiency is one of the characteristics of BS; however, the immunodeficiency is not well described and the underlying mechanism is not clear. A long-term follow-up of two BS patients showed repeated prolonged middle-ear infections and upper respiratory tract infections several times per year from 2 till 14 years of age; after which, the frequency of infections decreased [40]. In our cohort, two patients suffered from pneumonia, which is a common infection among BS patients [1]. However, most other infections were minor and did react well on antibiotics, and no uncommon infections were noticed. So, although the infections are not severe, infections are more common in BS patients compared to healthy controls [1]. Therefore, the question remains what causes this increased susceptibility for infections in these patients.

The longitudinal data of our cohort showed subnormal levels of serum immunoglobulin levels, especially for IgG and IgM. This decrease in serum immunoglobulin levels can either be explained by an intrinsic B cell defect or by a defect in stimulation of B cells by CD4+ T cells. In this cohort, the naïve B cell subsets were in the normal range, but the absolute numbers of natural effector B cells and memory B cells were significantly lower than the age-matched controls, which might suggest a maturation defect. Additionally, the CD4+ T cells were decreased. Combined, these two factors might contribute to increased numbers of infections observed in the BS patients.

BLM is involved in DNA repair and may have a role in the DNA repair-dependent processes during lymphoid development. Previous studies have shown that BLM does not have a role in V(D)J recombination [23, 41, 42], and we also did not observe differences in V, D, and J gene usage, and the number of deletions, palindromic (P)-nucleotides, and non-templated (N)-nucleotides (data not shown). The role of BLM in SHM has not been studied extensively. A study in a small number of rearrangements (12 IGHV sequences obtained from two BS patients) showed normal frequency of SHM and distribution of transition and transversion mutations in the BS patients [43]. Based on these results, Sack et al. conclude that de immunoglobulin hypermutation is normal in BS and that BLM plays no significant role in the process of SHM. In this study, we showed in a large number of rearrangements obtained from six BS patients low but normal frequency of SHM and normal SHM patterns. Together, these data suggest that BLM is not essential for SHM.

In mice models, there is no crucial role for BLM in the mechanism of CSR identified [23]. In this study, we showed that the memory B cells were low in three of the six BS patients, and the serum immunoglobulins levels were subnormal. The IGHG transcripts in humans showed particularly Cg3 and Cg1 gene usage with an increased use of short microhomology in the switch regions. This suggests that B cells in BS patients switch less to the more downstream Cg2 and Cg4 constant genes. Switching to these more downstream constant genes happens during the course of an immune response and requires CD4+ T cell stimulation [44, 45]. This indicates that the process of class switch recombination is disturbed in BS patients, which can either be caused by the low CD4+ T cells or an intrinsic B cell defect. Previous studies have shown that lymphocytes of Blm-deficient mice and BS patients (including P4, P5, and P6) have reduced proliferation capacity, which likely also contribute to reduced switching to the more downstream constant genes since these processes are dependent on B cell proliferation [23, 35, 36, 46, 47].

However, the immunodeficiency cannot be fully explained by an impaired CSR, because IgM is also produced at subnormal level, which is independent of CSR.

T cells in the BS patients were low in absolute numbers and percentages, for both CD4+ and CD8+. It is most likely that the process is already disturbed in the thymus since all T cell subsets are reduced compared to the HC. This suggests a possible role of BLM in the development of T cells. This was also earlier demonstrated in mice models, where mice with a conditional knockout of Blm in the T cell lineage have severely reduced thymocyte numbers [46]. The reaction of human T cells on phytohemagglutinin lymphocyte stimulation is disturbed and also stimulation by pokeweed mitogen is mostly decreased, which is an indication of less growth of B and T cells [35, 36]. Our results showed a relative increase of effector memory T cells compared to naïve and central memory T cells. So far, we can conclude that there is a disturbance in the development of T cells, which results in lower T cell levels, as is shown in our patient series. This reduced number of T cells could explain the low number of immunoglobulins and memory B cells, as CD4+ T cells stimulate SHM, CSR, and B cells to produce immunoglobulins.

## Conclusion

We showed BS patients suffer from relatively mild infections, which might be explained by the subnormal level of T, B, and NK cells and immunoglobulins. The B cell repertoire data on SHM, subclass distribution, and antigen selection of the B cells showed that the BS patients did not have great differences, but they clearly deviated from HC. Most importantly, despite the multiple disruptions, the immunodeficiency has a relatively mild character and functions in the laboratory and clinically at an acceptable level.

## Electronic supplementary material

Supplemental Table 1 (DOCX 27 kb)

Supplemental Figure 1 Serum immunoglobulin levels. The serum immunoglobulin levels of IgG1, IgG2, IgG3 and IgG4 over time in the 6 BS patients. (DOCX 705 kb)
