# Supplementary material for: Immunodeficiency in Bloom’s Syndrome
Source: J Clin Immunol. 2017 Nov 2;38(1):35–44. doi: 10.1007/s10875-017-0454-y (PMC5742600; doi:10.1007/s10875-017-0454-y)
Supplement: Supplementary file 1 — (DOCX 27 kb) [file 10875_2017_454_MOESM1_ESM.docx]

**Supplemental Table 1. The number of unique IGHA and IGHG transcripts for the controls and BS patients.**

|  | AGE | IGHA | IGHG |
| --- | --- | --- | --- |
| NWK64 | 7 | 111 |  |
| NWK61 | 8 | 73 |  |
| NWK66 | 9 | 96 | 65 |
| NWK57 | 14 | 223 | 395 |
| NWK42 | 15 | 136 | 174 |
| NWK5 | 15 | 129 | 58 |
| NWK297 | 31 | 1089 | 598 |
| NWK382 | 34 | 144 | 198 |
| perio34 | 35 | 1053 | 784 |
| perio37 | 36 | 576 | 1469 |
| NWK237 | 37 | 905 | 494 |
| NWK379 | 43 | 169 | 181 |
| NWK380 | 43 |  | 92 |
| NWK299 | 47 | 1332 | 630 |
| NWK378 | 49 | 354 | 540 |
| NWK377 | 55 | 917 | 469 |
| P1 | 9 | 380 | 353 |
| P2 | 12 | 603 | 342 |
| P3 | 12 | 701 | 284 |
| P4 | 35 | 654 | 466 |
| P5 | 37 | 691 | 413 |
| P6 | 46 | 699 | 473 |
