# Supplementary figures and images for: Immunodeficiency in Bloom’s Syndrome
Source: J Clin Immunol. 2017 Nov 2;38(1):35–44. doi: 10.1007/s10875-017-0454-y (PMC5742600; doi:10.1007/s10875-017-0454-y)

**Supplemental Figure 1**

**
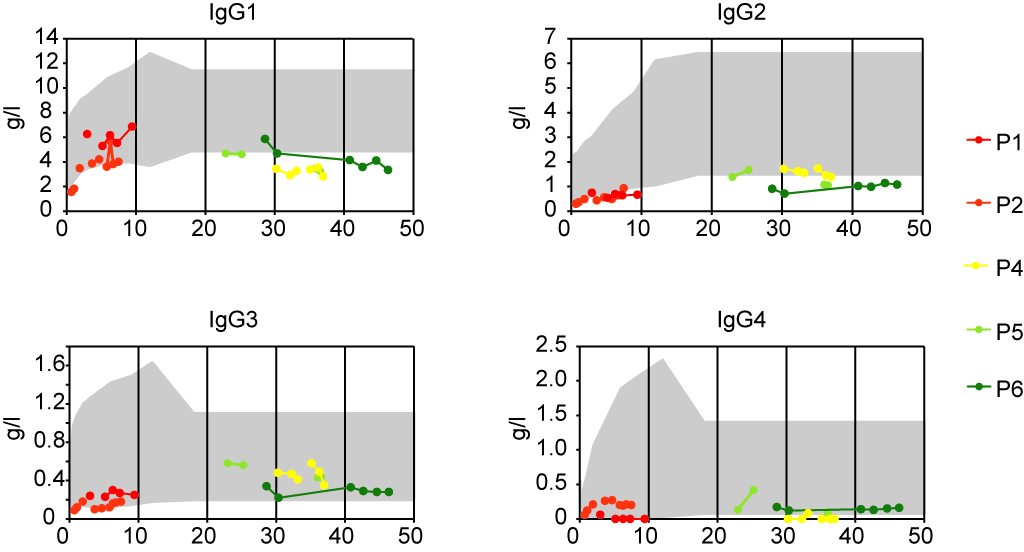
**

Supplement: Supplementary file 2 — Serum immunoglobulin levels. The serum immunoglobulin levels of IgG1, IgG2, IgG3 and IgG4 over time in the 6 BS patients. (DOCX 705 kb) [file 10875_2017_454_MOESM2_ESM.docx]
